# Neural specialization for speech in the first months of life

**DOI:** 10.1111/desc.12151

**Published:** 2014-02-27

**Authors:** Sarah Shultz, Athena Vouloumanos, Randi H Bennett, Kevin Pelphrey

**Affiliations:** 1 Department of Psychology, Yale UniversityUSA; 2 Department of Psychology, New York UniversityUSA; 3 Yale Child Study Center, Yale School of MedicineUSA

## Abstract

How does the brain’s response to speech change over the first months of life? Although behavioral findings indicate that neonates’ listening biases are sharpened over the first months of life, with a species-specific preference for speech emerging by 3 months, the neural substrates underlying this developmental change are unknown. We examined neural responses to speech compared with biological non-speech sounds in 1- to 4-month-old infants using fMRI. Infants heard speech and biological non-speech sounds, including heterospecific vocalizations and human non-speech. We observed a left-lateralized response in temporal cortex for speech compared to biological non-speech sounds, indicating that this region is highly selective for speech by the first month of life. Specifically, this brain region becomes increasingly selective for speech over the next 3 months as neural substrates become less responsive to non-speech sounds. These results reveal specific changes in neural responses during a developmental period characterized by rapid behavioral changes.

## Research highlights

1. By 1 month of age, human cortical circuitry is specialized for processing speech
2. Specialization of left temporal cortex for speech entails a decreased response to non-speech sounds between 1 and 4 months.

## Introduction

Learning to communicate depends critically on the ability to selectively attend to communicatively relevant signals in the environment. Human neonates are born with behavioral biases for attending to speech compared with a variety of synthetic and altered non-speech sounds (Spence & Decasper, 1987; Vouloumanos & Werker, 2007). Interestingly, however, neonates do not show a preference for speech compared with rhesus monkey vocalizations, suggesting that neonates’ listening biases may be relatively broadly tuned, encompassing a range of biological sounds, including conspecific and heterospecific vocalizations. These biases become attuned over the next few months of life, with 3-month-olds preferring speech over other biological sounds like rhesus vocalizations (Vouloumanos, Hauser, Werker & Martin, 2010) and human non-speech sounds like communicative vocalizations (e.g. laughter) and non-communicative vocalizations (e.g. coughs) (Shultz & Vouloumanos, 2010). These initial biases and eventual attunement to speech may be an adaptive mechanism, ensuring in-depth processing of speech and specialization of developing cortical circuitry. Although adults show specialized neural circuitry for processing speech relative to non-speech sounds (Binder, Frost, Hammeke, Cox, Rao & Prieto, 1997; Poeppel & Hickok, 2004; Shultz, Vouloumanos & Pelphrey, 2012; Vouloumanos, Kiehl, Werker & Liddle, 2001), the neural circuitry underlying the development of the human bias for speech during the critical behavioral changes in the first few months of life is poorly understood.

To the extent that neural circuitry is specialized for processing speech in early infancy, this neural specialization may be driven by any of several different properties of speech. Speech can be described on many levels, from its physical characterization as a collection of broadband frequencies that change over time, to its features that distinguish speech from other biological sounds (for instance, speech is produced by a human source, and more specifically, it is produced by the vocal tract). Prior studies examining the neural specialization for speech in early infancy did not contrast speech directly with biological sounds. Speech was contrasted with manipulated speech, which provided synthetic acoustic controls (Dehaene-Lambertz, Dehaene & Hertz-Pannier, 2002; May, Byers-Heinlein, Gervain & Werker, 2011; Perani, Saccuman, Scifo, Anwander, Spada & Baldoli, 2011; Peña, Maki, Kovačić, Dehaene-Lambertz, Koizumi, Bouquet & Mehler, 2003; Sato, Hirabayashi, Tsubokura, Kanai, Ashida, Konishi, Uchida-Ota, Konishi & Maki, 2012), or was combined with other vocalizations (Grossmann, Oberecker, Koch & Friederici, 2010); only one study contrasted speech with biological non-speech vocalizations, but the infants were 4 months old, past the age of behavioral specialization for speech (Minagawa-Kawai, Van der Lely, Ramus, Sato, Mazuka & Dupoux, 2011). As a result, the selectivity and specific developmental changes in cortical regions for processing speech compared to biological sounds that share important properties with speech in early infancy remains unknown.

In this paper, we address two specific aims: first we establish which regions respond to speech compared with other biological non-speech sounds such as heterospecific vocalizations and human non-speech sounds in early infancy. Specifically, we examine how broadly or finely tuned the neural specialization for speech might be by examining whether the regions that are responsive to speech are selective for speech or whether they are sensitive to all biological sounds. Second, within these speech-selective regions, we determine how this tuning or sensitivity for speech changes with age in the first few months of life. Given the sharpening of infant behavioral speech preferences from birth to 3 months, we predict that speech-sensitive brain regions undergo a process of specialization during this period. We consider two non-mutually exclusive hypotheses of cortical specialization: First, that cortical specialization for speech consists in neural substrates becoming increasingly responsive to speech. Second, that cortical specialization for speech consists in neural substrates becoming less responsive to non-speech sounds. This latter pattern would suggest that some neural substrates are already responsive to speech in early infancy, and further specialize by excluding sounds that are non-speech.

We used functional magnetic resonance imaging (fMRI) to investigate developmental changes in the specificity of the neural response to speech compared to biological non-speech sounds. During natural sleep, 24 1- to 4-month-old infants heard speech (adult-directed speech and infant-directed speech) and biological non-speech sounds produced by a human (communicative non-speech vocalizations, non-communicative vocalizations, human non-vocal sounds) or non-human (rhesus vocalizations) source.

## Method

### Participants

Thirty-eight 1- to 4-month-old infants were recruited into this study. Scans were attempted during natural sleep; no sedation was used. Twenty-seven of the 38 infants slept through the scanning session, yielding a data acquisition success rate of 71%. Data were not successfully acquired from 11 infants for the following reasons: the infant never fell asleep (4), the infant fell asleep but woke up while they were being prepared for the scanner (4), the infant woke up in the scanner before the start of the fMRI session (3). Of the 27 infants for whom data were acquired, three infants were excluded due to excessive motion during the functional scan.

The final sample consisted of 24 1- to 4-month-old infants (age range between 20 and 133 days, mean ± *SD* = 78 ± 38 days after birth, gestation corrected; 17 male, 7 female). Twenty-one of the infants were born at term, the remaining three were born at 36 weeks gestation. All infants had healthy deliveries and no history of ear infections or serious illness. As part of an ongoing longitudinal study, infants who participated at 1 to 2 months of age were invited to participate again at 3 to 4 months. As such, our final sample includes data acquired from three infants at two time points. All parents gave written informed consent. The study was approved by the Yale Human Investigations Committee.

### Stimuli and experimental design

Infants heard seven auditory conditions during natural sleep: adult-directed speech, infant-directed speech, human communicative non-speech vocalizations, human non-communicative vocalizations, human non-vocal sounds, rhesus macaque vocalizations, and sounds of water. Sounds of water were included in a contrast of all sounds compared to baseline but were not included in the contrast of speech compared with biological non-speech sounds (human non-speech and rhesus calls) because it is a non-biological signal. This stimulus set is similar to that used in an adult fMRI study of communicative auditory signals (Shultz *et al*., 2012) and a preferential looking paradigm assessing infant preferences for speech (Shultz & Vouloumanos, 2010) and has been well characterized in terms of its acoustic features. All sounds were sampled at 44,100 Hz, and equalized for mean intensity using PRAAT 5.1.07 (Boersma & Weenink, 2009). The sounds were concatenated into five 20-s sound files per sound category, each consisting of 11–15 tokens separated by 600–1000 ms of silence. The selection and ordering of tokens comprising the 20-s sound files was pseudorandom such that the same token was never played twice in a row.

Infant-directed speech consisted of 15 tokens of Japanese words spoken by three female native Japanese speakers. Words were spoken with slightly higher pitch and exaggerated pitch contour. We used Japanese speech to minimize the familiarity of specific speech tokens. Japanese is a mora-timed language that is perceptually distinct in prosodic and temporal organization from the predominantly stress-timed or syllable-timed languages spoken in these infants’ local community (Ramus, Nespor & Mehler, 2000). Importantly, infants under 6 months of age are highly sensitive to the rhythmic properties of speech and can discriminate utterances from different rhythmic classes (Mehler, Jusczyk, Lamsertz & French, 1988; Nazzi, Bertoncini & Mehler, 1998). None of the infants in the current study had been previously exposed to Japanese.

Adult-directed speech consisted of the same 15 tokens of Japanese words used in the infant-directed condition spoken by the same three female Japanese speakers. Words were spoken in a normal, neutral tone.

Human communicative vocal non-speech consisted of 15 tokens produced by three women: agreement (3), disagreement (3), disgust (3), inquiry (3), and laughter (3).

Human non-communicative vocal non-speech consisted of 15 tokens produced by three women: coughs (3), throat clearings (3), yawns (4), hiccups (3), and sneezes (2).

Human non-vocal sounds consisted of 15 tokens of three women walking on two surfaces: tile (7) and wood (8).

Rhesus macaque vocalizations consisted of 15 tokens produced by three free-ranging adult female rhesus macaques recorded in Cayo Santiago, Puerto Rico: grunts (2), coos (2), girneys (3), noisy screams (4) and arched screams (4). These calls differ from one another on valence and referential function (Hauser, 2000) and thus represent a wide range of rhesus vocalizations.

Water sounds consisted of 15 tokens downloaded from www.findsounds.com: running water (3), boiling water (3), water being poured (3), splashing water (3), and lake water (3).

Stimuli were presented in a block design with one sound category played per 20-s block. Each of the seven sound categories was played five times for a total of 35 blocks. Blocks were separated by a 12-s intertrial interval and presented in pseudorandom order such that the same sound category was never repeated more than twice in a row.

### Testing procedure and data acquisition

To reduce the noise level of the scanner, all infants were outfitted with earplugs (Mack’s Slim Fit Soft Foam Earplugs). Infant also wore MR-compatible headphones (Resonance Technology Inc.) to further reduce scanner noise and to play the stimuli. White noise, gradually increasing in volume, was played prior to the first scan to mask the onset of scanner noise. The white noise was played continuously throughout the scanning session (except during the fMRI task) to mask the start and end of scanner noise.

Data were acquired using a 3.0-T Siemens TIM TRIO scanner using a 32-channel head coil. Functional images were collected using an echo-planar pulse sequence (parameters: repetition time (TR) = 2 sec, echo time (TE) = 25 msec, flip angle = 60°, field of view = 220 mm, matrix = 64^2^, voxel size = 3.4 × 3.4 × 4 mm, 34 slices). Two sets of structural images were collected for registration: coplanar images, acquired using a T_1_ flash sequence (TR = 300 ms, TE = 2.46 ms, flip angle = 60°, field of view = 220 mm, matrix = 256^2^, 34 slices) and high-resolution images acquired using a 3-D MPRAGE sequence (TR = 2000 msec, TE = 2.96 msec, flip angle = 9°, field of view = 256 mm, matrix = 256^2^, voxel size = 1 × 1 × 1 mm, 160 slices).

An experimenter and a parent stood in the scanner room to observe the infant’s behavior at all times. The entire scanning procedure lasted less than 25 minutes and was stopped immediately if the infant awoke.

### Data analysis

#### Preprocessing

Data were preprocessed using the FMRIB Software Library (FSL, http://www.fmrib.ox.ac.uk/fsl). All images were skull-stripped using FSL’s brain extraction tool and supplemented by manual masking. The first six volumes (12 s) of each functional data set were discarded to diminish MR equilibration effects. Data were temporally realigned to correct for interleaved slice acquisition and spatially realigned to correct for head motion using FSL’s MCFLIRT linear realignment tool. To further reduce motion artifacts, individual blocks with a mean intensity of more than 3 standard deviations from the mean intensity of the scanning session were eliminated from further analyses. On average, 2.5 out of 35 blocks (range: 0–19) were removed per participant. Only one participant lost more than two of five blocks per condition. All other participants had at least three of five blocks of usable data per condition.

Images were spatially smoothed with a 5-mm full-width-half-maximum isotropic Gaussian kernel. Each time series was high-pass filtered (0.01 Hz cutoff) to eliminate low-frequency drift. Functional images were registered to structural coplanar images, which were then registered to high-resolution anatomical images and then normalized to a 3-month-old infant MRI template. The infant template was constructed from brain scans of 10 3-month-old infants acquired with a 3.0T scanner (Sanchez, Richards & Almli, 2012). A 3-month-old template was chosen because it is closest to the mean age of our sample (2.8 months, gestation corrected).

#### Statistical analysis

In fMRI studies the predicted BOLD response is typically expressed in terms of basis functions – which often include assumptions about the expected shape of the hemodynamic response function (HRF) – and compared to the collected time series. Each voxel is then characterized by a statistical measure that estimates how well the basis function models the variance over the course of the BOLD signal. Importantly, this technique may not be appropriate when the shape of the actual HRF differs from that of the assumed HRF. This concern is particularly relevant in the context of studying infant brain development where much debate exists about the exact nature, shape, timing parameters, and linearity of the HRF in infants (Seghier, Lazeyras & Huppi, 2006). As such we adopted a signal averaging approach (EventStats, Syam Gadde, Duke University) that does not rely on assumptions about the shape of the HRF.

The fMRI signal was converted to percent signal change relative to an inter-trial baseline and peri-event averages (epochs of the timeseries, time-locked to stimulus onsets for each condition) were computed for each condition of interest. A time-point by time-point *t*-statistic was calculated at each voxel for each subject. Group statistics were computed using a weighted *z*-test (Whitlock, 2005).

To correct for multiple comparisons, we used the AlphaSim program included in the Analysis of Functional NeuroImages (Cox, 1996). A minimum cluster size of 74 mm^3^ voxels was used to achieve a corrected significance of *p*<.05 as determined by a Monte Carlo simulation with our voxel-wise threshold of *p*<.05. A minimum cluster size of 20 mm^3^ voxels was used to achieve a corrected significance of *p*<.05 with a voxel-wise threshold of *p*<.01. A brain mask, created by combining the white matter and gray matter segmentation volumes associated with the infant template, was applied to all statistical maps. Clusters of statistically significant activation are described by their corresponding anatomical labels in a Harvard-Oxford stereotaxic atlas designed for 3-month-old infants (Phillips, Richards, Stevens & Connington, 2012; Richards, Stevens & Connington, 2012a, 2012b).

## Results

### Activation to sound

**Figure 1:**
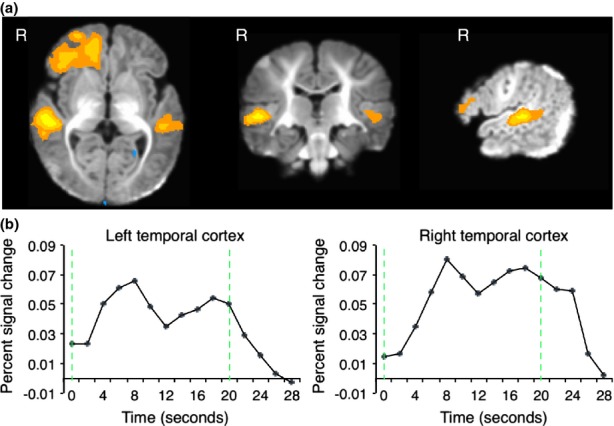
(a) Activation evoked to all sound conditions relative to baseline (voxel p < .01, cluster p < .05, corrected) in 1- to 4-month-old infants. The color ranges from p = .01 to p = 0. (b) BOLD percent signal change for all sound trials averaged across voxels within left and right temporal regions from the all sounds versus baseline contrast. Green dashed lines indicate stimulus onset and offset. In both left and right cortices activation peaks 8 seconds after stimulus onset.

We first conducted a whole-brain contrast of all sounds compared with baseline to verify that our experimental design and analysis approach were sensitive enough to capture a response to sounds in primary auditory cortex. The whole-brain analysis of all sounds compared with baseline (see Figure1a; see also Table1) revealed significant bilateral activation in the posterior division of the middle and superior temporal gyrus, and Heschl’s gyrus, a pattern of activation consistent with previous studies of auditory processing in infancy (Blasi, Mercure, Lloyd-Fox, Thomson, Brammer, Sauter, Deeley, Barker, Renvall, Deoni, Gasston, Williams, Johnson, Simmons & Murphy, 2011; Dehaene-Lambertz *et al*., 2002). Widespread activation to sound was also observed in right frontal cortex extending through the frontal pole, frontal orbital cortex, the inferior frontal gyrus (including pars opercularis and pars triangularis), and the middle frontal gyrus. These results confirm that our data collection and analysis techniques were sensitive enough to detect auditory activation in these naturally sleeping infants.

**Table 1:** Anatomical labels for each active cluster (voxel p < .01, cluster p < .05, corrected) for the All Sounds > Baseline contrast. Anatomical labels are as indicated by a Harvard-Oxford Cortical Atlas normalized to a 3-month-old template.

All Sounds > Baseline	
Region	Number of Voxels
**Right Frontal Cortex**	14335
Frontal pole	
Frontal orbital cortex	
Middle frontal gyrus	
Pars opercularis	
Pars triangularis	
**Right Temporal Cortex**	2243
Middle temporal gyrus, posterior division and temporoccipital part	
Superior temporal gyrus, posterior division	
Heschl’s gyrus	
**Left Temporal Cortex**	874
Middle temporal gyrus, posterior division	
Superior temporal gyrus, posterior division	
Heschl’s gyrus	
Left Frontal Pole	48

We also examined the shape of the hemodynamic response to sound (see Figure1b). Average percent signal change time courses from the activated voxels in left and right temporal cortices revealed that the hemodynamic response peaks approximately 8 seconds after stimulus onset followed by another smaller peak approximately 18 seconds after stimulus onset, a pattern that is strikingly similar to that reported previously in 2- to 3-month-old infants (Dehaene-Lambertz *et al*., 2002). Given that the response to sound was strongest 8 seconds after stimulus onset, all subsequent analyses were conducted on responses observed at that time point.

### Activation to speech versus biological non-speech

**Figure 2:**
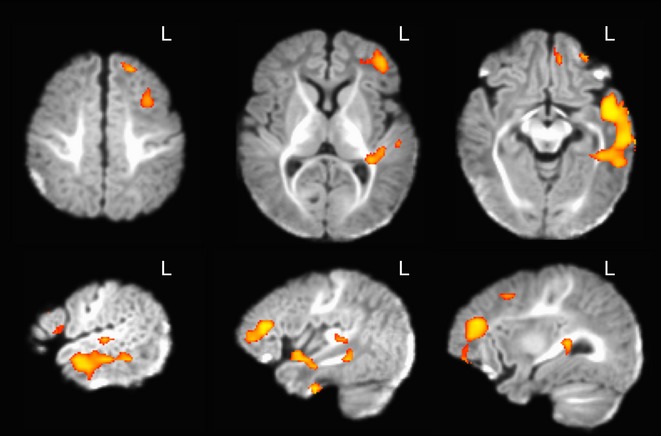
Axial and sagittal views of activation evoked to speech compared to biological non-speech sounds (voxel p < .05, cluster p < .05, corrected) in 1- to 4-month-old infants. The color ranges from p = .05 to p = 0.

A whole-brain contrast of all speech versus all biological non-speech sounds (Figure2; see also Table2) revealed significant activation in large clusters of left temporal and left frontal cortex. Activation in left temporal cortex encompassed the anterior and posterior divisions of the superior temporal gyrus, the anterior, posterior, and temporoccipital parts of the middle temporal gyrus, the anterior, posterior, and temporoccipital parts of the inferior temporal gyrus, Heschl’s gyrus, the posterior division of the temporal fusiform cortex, and the temporal pole. Activation in left frontal cortex encompassed the frontal pole and the inferior frontal gyrus (including pars triangularis). Additional clusters of activation are reported in Table2. These findings suggest that 1- to 4-month-old infants have neural structures that respond selectively to speech compared with biological non-speech sounds.

**Table 2:** Anatomical labels for each active cluster (voxel p < .05, cluster p < .05, corrected) for the Speech > Biological Non-Speech contrast. Anatomical labels are as indicated by a Harvard-Oxford Cortical Atlas normalized to a 3-month-old template.

Speech > Biological Non-Speech	
Region	Number of Voxels
**Left Temporal Cortex**	4570
Superior temporal gyrus, anterior and posterior division Heschl’s gyrus	
Middle temporal gyrus, anterior, posterior, and temporoccipital part	
Inferior temporal gyrus, anterior, posterior, and temporoccipital part	
Temporal fusiform cortex, posterior division Temporal pole	
**Left Frontal Cortex**	3315
Frontal pole	
Inferior frontal gyrus, pars triangularis	
Left frontal orbital cortex and temporal pole	199
Left middle frontal gyrus	145
Left superior temporal gyrus, posterior division	127
Left superior parietal lobule and postcentral gyrus	124
Left cerebellum	206
Left frontal pole	97
Left inferior frontal gyrus, pars triangularis and pars opercularis	92
Left middle frontal gyrus	79

**Figure 3:**
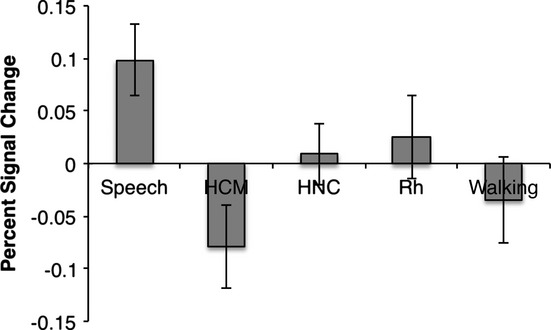
Percent signal change, averaged across all voxels within speech-sensitive left temporal cortex, in response to each sound category in 1- to 4-month-old infants. The difference in response to speech compared with human communicative non-speech vocalizations (HCM) and sounds of walking is significant (all ps < .05). The difference in response to speech compared with human non-communicative non-speech vocalizations (HNC) and rhesus calls (Rh) was marginally significant (p = .081 and p = .062, respectively).

While human communicative vocal non-speech, human non-communicative vocal non-speech, rhesus macaque vocalizations, and human non-vocal sounds are all biological non-speech signals, we recognize that these non-speech sounds vary in the extent to which they share other key properties with speech. For instance, speech is a signal produced by a human source (as are human non-speech vocalizations and human non-vocal sounds) and is produced by the vocal tract (as are human non-speech vocalizations and rhesus macaque vocalizations). As such, it is possible that regions identified in the contrast of speech versus all biological non-speech sounds may also show some selectivity for all human or all vocal sounds, rather than showing selectivity for speech *per se*. To examine this possibility, we averaged the percent signal change, for each stimulus category, across all voxels within the region of left temporal cortex identified in the speech versus all biological non-speech contrast (see Figure3). We chose to examine the response to each stimulus category in this region of left temporal cortex because of previous findings indicating that speech processing is localized to regions of left temporal cortex in older infants and adults (Binder *et al*., 1997; Dehaene-Lambertz *et al*., 2002) and the current corroborating results. As expected, a repeated-measures ANOVA revealed a main effect of condition (*F*(4, 92) = 3.03, *p*<.05) (this is expected because this region was defined by the speech > biological non-speech contrast). Paired samples *t*-tests revealed that the response to speech in this region was significantly greater than the response to human communicative non-speech vocalizations (*t*(23) = 3.23, *p*<.01) and sounds of walking (*t*(23) = 2.08, *p*<.05). The increase in response to speech was marginally significant compared to rhesus (*t*(23) = 1.96, *p*=.062) and human non-communicative non-speech vocalizations (*t*(23) = 1.82, *p*=.081). These results suggest that this region is indeed specialized for speech *per se*, rather than for all human or all vocal sounds.

### Age-related differences in speech-sensitive left temporal cortex

**Figure 4:**
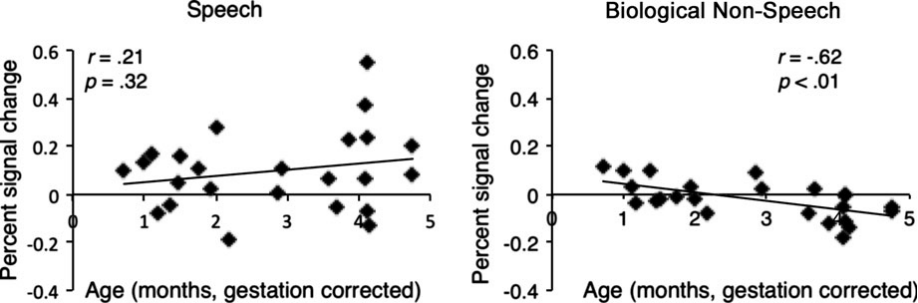
Age-related changes in the percent signal change in response to speech and biological non-speech sounds (averaged across all voxels within the region of left temporal cortex identified in the speech versus biological non-speech contrast). There is a significant negative correlation between age and percent signal change in response to biological non-speech sounds.

To investigate whether speech-sensitive regions become increasingly specialized for speech with age, we examined correlations between the percent signal change in response to speech or biological non-speech sounds (averaged across all voxels within the region of left temporal cortex identified in the group-level speech versus biological non-speech contrast) and age. A large negative correlation was observed between age and the response to biological non-speech (*r* = −.62, *p*=.001) (Figure4), indicating that this region becomes less responsive to biological non-speech with age. The correlation between age and the response to speech was not significant (*r*=.21, *p*=.32).

## Discussion

Using fMRI, we observed a strongly left-lateralized response to speech over biological non-speech sounds in infants as young as 1 month old. The difference in activation within left temporal cortex for speech over biological non-speech increased with age over the next 3 months of life; specifically the response to speech in this region was sustained while the response to biological non-speech decreased. Thus, the specialization of developing cortical circuitry for speech between 1 and 4 months entails the attenuation of responses to biological non-speech sounds. This study is the first to report patterns of developmental change in the neural specialization for speech over the first few months of life.

This cortical specialization for speech is highly consistent with behavioral results indicating that neonates’ listening biases, encompassing both conspecific and heterospecific vocalizations, become attuned over the next few months of life, yielding a preference for speech over other biological sounds by 3 months. This perceptual narrowing and cortical specialization may reflect an experience-expectant process whereby cortical circuitry and listening preferences that are initially broadly tuned become increasingly refined with exposure to speech (Johnson, 2001; Kuhl, 2004; Leppänen & Nelson, 2009). While the precise neural mechanisms underlying this process are unknown, it has been proposed that experience-expectant mechanisms may involve the generation of an excess of synaptic connections, with experiential input subsequently determining which synapses will become elaborated or sustained and which will be lost (Greenough, Black & Wallace, 1987). Indeed, the months after birth are a time of rapid synaptic proliferation, with synaptic density in primary auditory cortex reaching a maximum at 3 to 4 months of age, followed by a period of gradual pruning (Huttenlocher & Dabholkar, 1997). The current data suggest that neural specialization between 1 and 4 months may be characterized by selective pruning, given the sustained response to speech coupled with the attenuation of neural responses to other biological non-speech sounds. Interestingly, however, 3- to 4-month-olds showed a numerically larger response to speech in left temporal cortex compared to 1- to 2-month-olds. Although this difference was not significant it may suggest that there is indeed some strengthening of the response to speech in left temporal cortex from 1 to 4 months. While a significant increase in response to speech with age was not observed in the present study, it is also possible that such an increase may occur between birth and 1 month of age.

Our study adds to the handful of neuroimaging studies examining speech perception in the first few months of life. Although prior neuroimaging studies have not been completely consistent, most corroborate the behavioral data showing specialized neural systems in the left hemisphere for speech processing in 3- to 4-month-olds (Dehaene-Lambertz, Montavont, Jobert, Allirol, Dubois, Hertz-Pannier & Dehaene, 2010; Dehaene-Lambertz *et al*., 2002; Minagawa-Kawai *et al*., 2011). But data before 3 months are mixed, with some studies showing left hemisphere activation for continuous native speech over backward speech and silence (Peña *et al*., 2003) and for continuous native speech over backward native speech and non-native speech (Sato *et al*., 2012), others showing bilateral temporal and inferior frontal activation for continuous native speech over silence (Perani *et al*., 2011), and still others showing equal activation for continuous native forward and backward speech (May *et al*., 2011). One source of heterogeneity is likely the use of different neuroimaging methodologies, fMRI versus functional near-infrared spectroscopy (fNIRS), which can yield different results (compare the localization differences in the fMRI and fNIRS results using the same stimuli in the same aged infants (Blasi *et al*., 2011; Lloyd-Fox, Blasi, Mercure, Elwell & Johnson, 2011)). Another source of variability may be the nature of the contrasts tested or particular characteristics of the stimuli used. For instance, the study reporting bilateral activation to speech compared with non-speech sounds contrasted speech with silence (Perani *et al*., 2011), whereas all studies reporting a left-lateralized speech response contrasted speech with another sound (typically non-native speech or backward speech). Further, the only study reporting no specialized response to forward compared with backward speech used low-pass filtered speech samples (May *et al*., 2011), whereas other studies used unfiltered speech.

While the current study indicates that 1- to 4-month-olds show a left-lateralized neural response to speech compared to biological non-speech sounds similar to adults, there are also some interesting differences compared with neural activation to speech in adults. Consistent with adult studies, we observed left-lateralized activation in the superior, middle, and inferior temporal gyrus and in regions of the inferior frontal gyrus (Binder *et al*., 1997). However, we also observed activation in areas that are not typically reported in adults, such as the postcentral sulcus, and superior parietal lobule (see Table2). This may suggest that speech processing in infancy engages a wider range of brain regions and becomes increasingly localized over development (Johnson, 2001). Finally, we did not observe activation in the angular gyrus, a region that is selective for speech in both adults (Binder *et al*., 1997) and in older infants (Dehaene-Lambertz *et al*., 2002), indicating that the angular gyrus may not yet be selective for speech compared to other biological non-speech sounds in the first few months of life.

In conclusion, the present study contrasts brain responses to speech with other biological non-speech sounds during a period of developmental change that is characterized by a sharpening of infant listening preferences for speech. The response to biological non-speech in left temporal cortex decreased with age, resulting in increased specialization for speech, a process that may be both a cause and a consequence of the tuning of infants’ preference for speech.
